# Frequency-resolved optical gating measurement of ultrashort pulses by using single nanowire

**DOI:** 10.1038/srep33181

**Published:** 2016-09-09

**Authors:** Jiaxin Yu, Feng Liao, Fuxing Gu, Heping Zeng

**Affiliations:** 1 Shanghai Key Laboratory of Modern Optical System, Engineering Research Center of Optical Instrument and System (Ministry of Education), University of Shanghai for Science and Technology, Shanghai 200093, P. R. China; 2 State Key Laboratory of Precision Spectroscopy, East China Normal University, Shanghai, 200062, P. R. China

## Abstract

The use of ultrashort pulses for fundamental studies and applications has been increasing rapidly in the past decades. Along with the development of ultrashort lasers, exploring new pulse diagnositic approaches with higher signal-to-noise ratio have attracted great scientific and technological interests. In this work, we demonstrate a simple technique of ultrashort pulses characterization with a single semiconductor nanowire. By performing a frequency-resolved optical gating method with a ZnO nanowire coupled to tapered optical microfibers, the phase and amplitude of a pulse series are extracted. The generated signals from the transverse frequency conversion process can be spatially distinguished from the input, so the signal-to-noise ratio is improved and permits lower energy pulses to be identified. Besides, since the nanometer scale of the nonlinear medium provides relaxed phase-matching constraints, a measurement of 300-nm-wide supercontinuum pulses is achieved. This system is highly compatible with standard optical fiber systems, and shows a great potential for applications such as on-chip optical communication.

Ultrashort pulse lasers have made great progress since its emergence. As an important research field, they have been motivated by the rapid advance of various applications ranging from optical imaging and spectroscopy, to laser processing and optical communication^1^,^2^. Their dramatic development and extensive applications urge us to fully and intactly characterize an ultrashort pulse. Among all the techniques, frequency-resolved optical gating (FROG) is a common and effective approach. Conventional FROG system requires a nonlinear optical (NLO) material in bulk in order to gain abundant signal photons for detection, and as a trade-off, the setup is always elaborate and presents a considerable complexity, especially for pulses like super-continuum^3^,^4^. Meanwhile, modern spectro/microscopy like coherent Raman spectroscopy, tip-enhanced spectroscopy, and multi-photon microscopy have got emerging interests towards higher resolution in temporal, spectral and spatial domains. Because of the sophisticated system, however, it is impossible to implement an *in-situ* measurement, and thus detailed information of the target site is still missing^5^,^6^,^7^.

Nano-scale materials have the inherent advantage on fine structure imaging and spectral analysis, since they can be employed as tracking material without perturbing the local environment. The usage of nanomaterial based FROG (nano-FROG) would relieve the phase-matching condition, and provide an avenue to monitor the nano-domain distortions in near field^8^,^9^,^10^,^11^. Exsiting nano-FROG methods, either via randomly dispersed nanoparticles or via nanoparticles attached to fiber taper, their FROG signal collections still rely on free-space optics. The loss by a series optical elements largely limits the sensitivity and reliability of the apparatus. Moreover, the NLO signal of these methods is mixed with fundamental wave in collection, so it requires optical filters to differentiate them. The irradiation detection further imposes a restriction in measuring broadband pulse when fundamental wave partly overlaps with signal in spectrum, and thus filters are not able to separate them. Besides, the variation and irregularity of the nanomaterials make them improbable to reproduce a consistent result among different measurements. More recently, transverse frequency conversion in semiconductor nanowires has been attracted lots of attentions because of its high conversion efficiency and low divergence angle^8^,^12^,^13^,^14^; although the feasibility of pulse measurement has been discussed, however, using this scheme to perform FROG measurement and retrieval of ultrashort and broadband pulses have not been reported yet. In this paper, we demonstrate a waveguide based FROG method with single nanowire (NW) as NLO material, through which the phase and amplitude of pulses are measured.

## Results

### NW synthesis and Characterization

**Figure 1:**
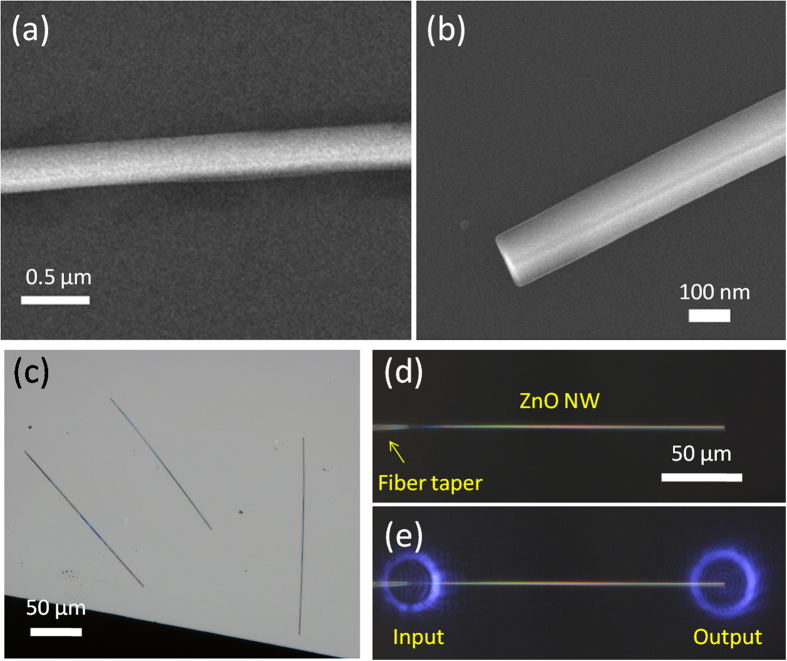
Characterization of as-synthesized ZnO NWs and optical guiding properties. (**a**) Scanning electron microscope image of a ZnO NW. (**b**) Enlarged SEM image of ZnO NW after fracture. (**c**) Bright-field optical microscope image of ZnO NWs deposited on a silicon substrate. (**d**) Light coupling approach in to a 180-μm-long ZnO NW using a fiber taper. (**e**) Optical microscope image of guiding a 1064-nm-wavelength laser.

Here we chose the zinc oxide (ZnO) as the NLO material, which is a typical II-VI semiconductor with moderate absorption loss and wide band gap (3.37 eV, 368 nm)^15^. ZnO NW used here was synthesized via a vapor-liquid-solid (VLS) process^16^. Figure 1(a) gives the scanning electron microscopy (SEM) image of as-fabricated ZnO NW, showing the NWs with good smoothness and uniformity; to obtain high reflection end faces, we use a tungsten probe to fracture the NW by micro-manipulation^13^,^14^, and the endface image is shown in Fig. 1(b). Before manipulation, the ZnO NWs were removed from the growth substrate and deposited on a low refractive-index MgF_2_ substrate, and the typical optical micrograph was shown in Fig. 1(c). The as-fabricated NWs were all around 200 ~ 400 nm in diameter and the lengths can be up to several hundreds of micrometers. By means of butt-coupling technique under a microscope^13^,^14^, ZnO NW can be manipulated and placed onto a a silica optical fiber taper, so that the light can be efficiently transferred into the NW. Figure 1(d) shows the micrograph of a random fiber taper-NW couple, and its optical guiding behavior can be examined by launching a 1064-nm-wavelength continuous-wave (CW) laser as shown in Fig. 1(e), from which we can find there is no scattering spots on the NW, and its conducting feature is good.

### Experiment setup

**Figure 2:**
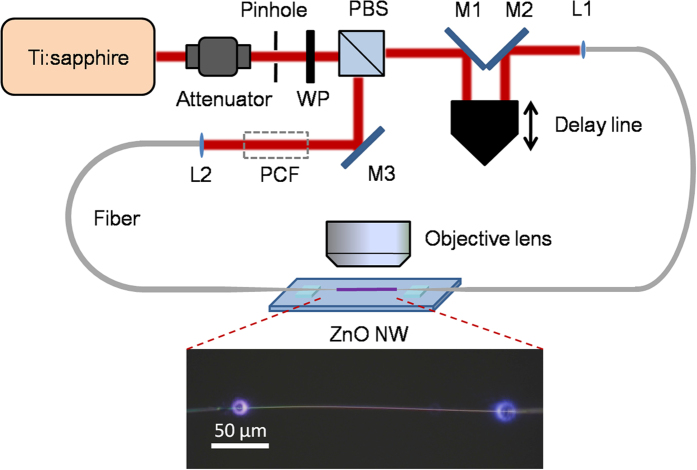
Experiment setup of FROG. WP: wave plate; L1 and L2: focal lens; M1-M3: total-reflection mirror. Inset: Bright-field optical microscope image of a suspended ZnO NW with 810 nm input from both ends.

The schematic diagram of the measurement setup is illustrated in Fig. 2. A Ti:Sapphire mode-locked laser with central wavelength at 810 nm (80 MHz, ~70 fs, Spectra-Physics) was used here. After going through an isolator and a pinhole, the laser beam was split into two halves by a wave plate and a polarization beam splitter (PBS). The input power of the 810 nm pulsed light was ~10 mW for each arm and the light transfer efficiency was measured to be ~60% for single taper-NW coupling. One beam was used as target pulse, and the other beam as gate pulse was introduced into a retro-reflector, by which the length of optical path can be tuned directly. Two beams were individually coupled into two single-mode fibers (112 cm long each) whose ends were tapered to microfibers. Both tapered microfibers were suspended above a MgF_2_ microchannel with ~200 μm in seperation. A 245-μm-long, 310-nm-diameter ZnO NW was coupled with them via micro-manipulation under optical microscope^13^,^14^,^17^. The angle and position of both tapers can be carefully adjusted to maintain an optimal input coupling efficiency. The surface emitted signal from the NW was collected by a objective lens, and then filtered and directed into a CCD (DS Ri1, Nikon); part of the signal was reflected by a neutral beam-splitter (45/55) into a spectrometer (QE65 pro, Ocean Optics).

**Figure 3:**
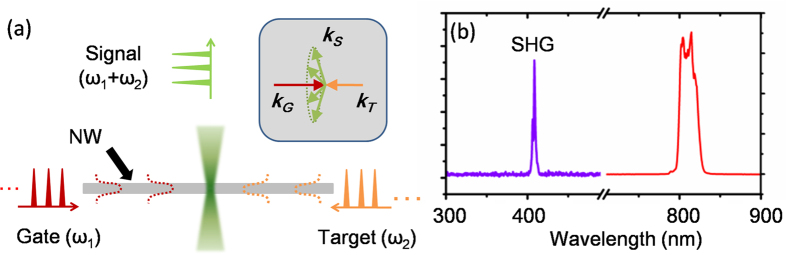
Frequency conversion by a ZnO NW. (**a**) Schematic of SFG generated by gate and target pulses in a NW. Inset: phase-matching condition for SFG. (**b**) Measured spectrum of fundamental and SH wave.

The mechanism of surface emitted sum frequency (SF) signal generated in a NW is sketched in Fig. 3(a), and we take second harmonic (SH) process as a special case of SF with two identical inputs. Briefly, two series of pulses are injected from each end of the NW and counter propagate in it. By tuning the delay line, pulses from opposite directions collide within the NW^8^,^14^. As required by phase-matching conditions, the nonlinear optical emission will be generated transversely as shown in Fig. 3(a) inset. With two series of 810 nm pulses injected from both ends, the spectra of signal can be detected perpendicularly. As shown in Fig. 3(b) that the signal was at the half-wavelength position of fundamental light with several nanometers in width, which confirmed it to be SH signal. Since the optical path interval between two neighboring pulses is in the scale of several meters, only one pair of pulses can be in the NW at the same time. Besides, from our result, the SH signal generated by one pulse beam through the birefringent process was tens of times weaker than the SH signal generated by two pulse correlating, which allowed for the measurement of pulses with very low energy.

### Auto-correlation

**Figure 4:**
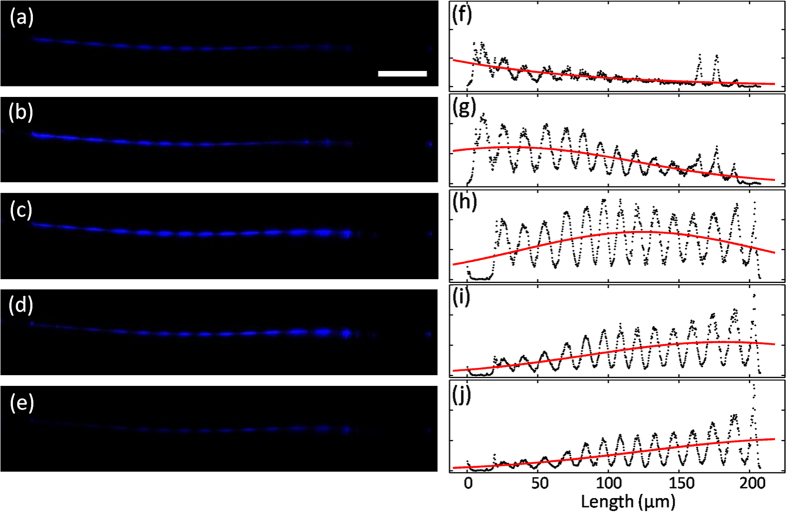
Counter-propagating pulses. (**a**–**e**) Optical microscope images of SHG with different delay in optical path. Scale bar, 50 μm. (**f**–**j**) Corresponding measured (black dots) and fitted (red line) intensity profile of (**a**–**e**).

To verify the feasibility of using single NW for pulses characterization, we first study the pulses colliding process in the NW. Figure 4(a–e) were SH images recorded at around 405 nm during the pulse series optically gated by itself. The target pulses came from right and the gate controlled by retro-reflector came from left. It can be seen that the SH signal was first generated from left end of ZnO NW [Fig. 4(a)], become stronger with the pulses moving in [Fig. 4(b)], then reached its maximum in Fig. 4(c) and disappeared from right end of NW finally [Fig. 4(d,e)]. The corresponding position-dependent SH intensity curves of Fig. 4(a–e) were plotted in Fig. 4(f–j) in black dots by integrating the intensity of image pixels, and the Y-axis of each plot were altered to form a better contrast. The periodical pattern was originated from the guided modes of the counter-propagating waves. As proved in our previous study^13^,^14^, the guided modes in NW depend on the diameter of NW, the relative position and the angle between the fiber taper and the NW. And the guided modes would further influence the SH emission. The spatial distributions in transverse SH emission are determined by the relationship of *I*^*SH*^ ∝ cos^2^ (Δ*β*z), where Δ*β* represents the propagation constant difference between the encountered guided modes. So the SH images of NW could manifest an oscillation behavior when Δ*β* ≠ 0. Nevertheless, the SH spectrum would not be influenced by the change of patterns. The red line for each panel in Fig. 4(f–j) plotted the Gaussian fitted profiles in order to make the movement of the intensity peak more obvious.

### FROG measurement of 22-nm-wide pulses

**Figure 5:**
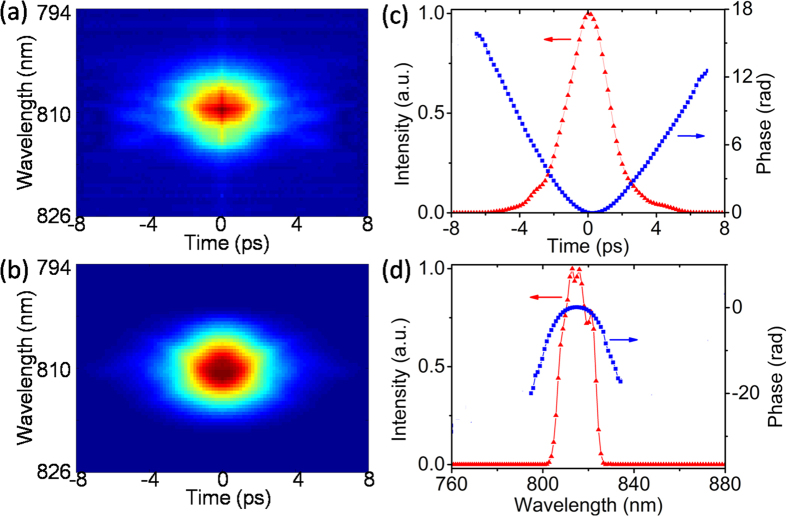
FROG trace of 810 nm pulses. (**a**) Measured and (**b**) retrieved trace. Electric field intensity (red triangle) and phase (blue square) as a function of time (**c**) and of wavelength (**d**).

From the intensity pattern in Fig. 4, we could find that with continuous movement of the delay line, the SH maximum could move in and out of the NW. For a rough estimation of pulse duration, we observed the variation of intensity at an arbitrary position of NW [i.e. right in the middle of Fig. 4(f)], and marked the displacement of the delay line between its two half-maximum, which was ~150 μm. So the full width at half maximum (FWHM) of a pulse could be calculated by ~300 μm divided by light speed, which was in the scale of picoseconds in time profile. To operate a more precise pulse characterization, we measured the auto-correlating spectra of pulses counter-propagating through the NW. With a speed of 25 μm/step, the surface emitted SH signal could be scanned out. The pulse energy was about 50 pJ/pulse and a typical spectrum was taken with 1 s exposure time. Figure 5(a) shows the spectra collection in form of a 128 × 128 grid graph, in which 128 spectra were placed in chronological order, and the intensities of spectra were represented by colormap. Notice that the temporal index was transformed from free-space optical-length displacement of delay line. Based on the results, a retrieved FROG trace could be readily obtained by a method given in ref. 18 [Fig. 5(b)]. Briefly, the method involved a solution to two-dimentional phase-retrieval problem. Two constraint sets, nonlinear-optical set and the experimental data set, are to be satisfied by alternately iteratively projecting from an initial guess. The intersection of the two constraints will lead to a solution of pulse electric field with reliable temporal and spectral information. The measured and retrieved FROG traces in Fig. 5 are in good qualitative agreement with the G error of 0.0107, which could be elevated with a higher-resolution spectrometer. The pulse intensity and phase profile are also presented in both temporal and spectral domains, as shown in Fig. 5(c,d), respectively. The intensity profile in Fig. 5(c) indicates the pulse duration of 2.8 ps, much longer than the source output. The pulse broadening mainly occurred in two parts: the ~1.1 m-length fiber, and the ~10-mm-length tapered microfiber. For a rough estimation, we can expect the dispersion as ~118 ps/nm/km for an 810-nm pulse with 22-nm width in a 1-m-long single-mode fiber (SMF-28, Corning), which leads to pulse broadening of more than 2.8 ps^19^. While for the fiber taper, the dispersion in the 1-μm-diameter microfiber end was estimated around 400 ps/nm/km^20^. Assuming that the dispersion changes linearly in the taper, the pulse width should increase for another ~100 fs. Since the length of the ZnO NW is relative short (~200 μm), the pulse broadening imposed was neglected here. Therefore, in the process the pulse change should be ~2.8 ps, almost consistent with our measurement results.

### Cross-correlation FROG (XFROG) measurement of 300-nm-wide pulses

**Figure 6:**
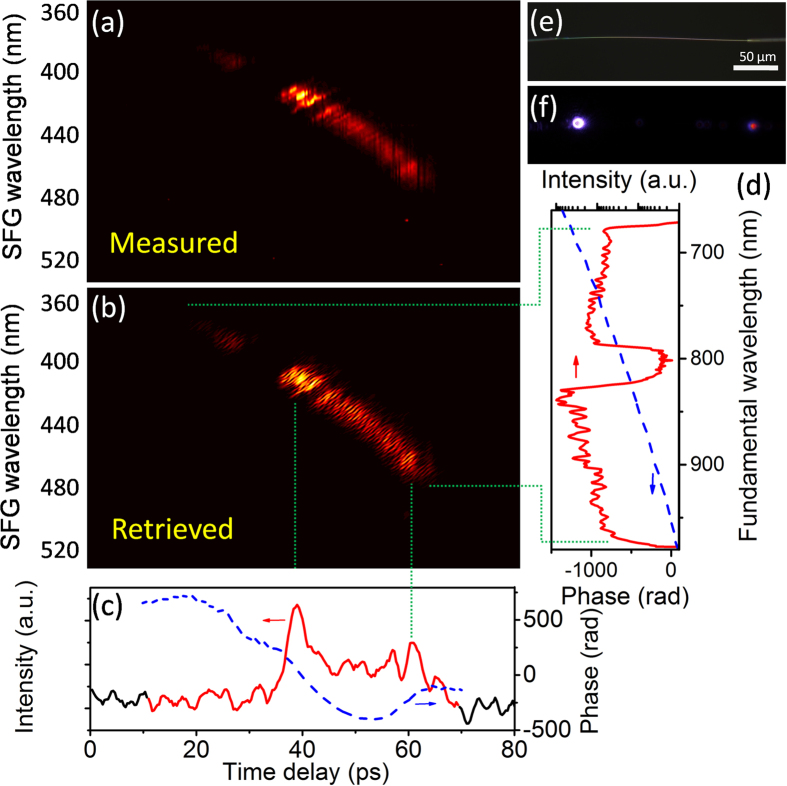
XFROG trace of super-continuum pulses. (**a**) Measured and (**b**) retrieved trace with its structure correlated with the temporal intensity (**c**) and excitation spectrum (**d**). (**e**) Bright-field optical microscope image of a suspended ZnO NW. Scale bar, 50 μm. (**f**) Dark-field image of the same NW with 810 nm input from left end while SC from right.

Since ZnO NW has broadband transmittance in the visible to NIR spectral region, we further operated cross-FROG to measure supercontinuum (SC) pulses on the same system. The SC was generated by adding a photonic crystal fiber (FemtoWHITE, Newport) into the path between M3 and L2 in Fig. 2. A norch filter (NF03–808E, Semrock) was used to block the fundamental wave portion in the SC, otherwise the signal from the broadband portion would be under low contrast. Besides, a long-pass filter (BLP01–664R, Semrock) was inserted to avoid the absorption of NW, which might induce undesired luminescence. After both filters, the SC power output from PCF was left for ~60 mW, which was 2.5 pJ/nm/pulse in average for overall 300-nm-wide band. For the XFROG measurement, the SC was gated by the 810 nm pump pulse. The delay between the SC and gate pulses was scanned in 300-fs steps, and a series of SF signal generated by SC and the gate pulses was resolved at each delay forming a 256 × 256 trace [Fig. 6(a)]. Using the same method as refered above^18^, the measure trace was further interpolated into a 4096 × 4096 trace for retrieval as required by the discrete Fourier transform relation, and the result was presented in Fig. 6(b). The G error was 0.0123, which was not as good as that in Fig. 5, it is because the complexity of pulses was largely increased in both time- and spectrum-bandwidths.

In Fig. 6(c,d), we have plotted the retrieved pulse intensity and phase profile in temporal and spectral domains after 20-point smoothing, indicating the SC pulses with a ~55-ps time span [Fig. 6(c)] and 670 nm ~ 970 nm in wavelength [Fig. 6(d)]. It is obvious that the SC pulses were largely broadened temporally even more than the pump pulses measured above. It is reasonable because its wavelength band was expanded to more than 300 nm in the PCF, and most of it stood in the same dispersion regime as it did for pump pulses. Therefore, the chirp became more serious in the conducting fiber compared to the 20-nm pump pulses, and led to higher pulse duration. Figure 6(e,f) present the images of NW without and with pulses imposing from each end of it (810 nm from left and SC from right). It could be noticed that almost no SH emission was generated by gate or target pulses themselves, which ensured a clean spectral background for pulse measurement.

## Discussion

In this work, we have implemented a nano-FROG method for ultrafast pulse characterization. Since the pulse modulation (stretching, compressing, etc.) by all the components have been involved and counted, the measured results of our method reveal the *in-situ* characteristics of the pulse, which means it could be a good solution to the measurement in fiber-based integrated systems. Compared with the previously reported FROG approaches, this fiber taper-NW coupling system exhibits several superiorities: (I) In our system, the target pulses are optically guided along NLO NW rather than spacially focused. Different from the free-space FROG, the majority of energy loss caused by the small duty ratio (the cross section ratio between the NW and focal spot), and by the scattering on the interface could be eliminated, which makes it more sensitive to low-energy pulses. More important, compared with the exsiting free-space nano-FROG methods, the light wave is guided along the NW, so the out-of-focus problem caused by wide-waveband dispersion can be avoided, permitting the measurement of SC pulses with broadband spectrum. While for the pulse disturbance from the waveguide system, it is predictable and thus can be compensated before measurement. (II) Since the signal was emitted from the NW surface, the direction of SF signal and input pulses are perpendicularly to each other, so the remnant fundamental wave will be spatially precluded. In most FROG approaches where SF and fundamental waves are propagating coaxially, optical filters are required to separate them spectrally. Compared with this, spatial separation by transverse emission has good advantage especially when a spectral overlap exists between fundamental and SF wave. Imagine the following situation, 800-nm gate pulses and SC pulses with 400 nm ~ 1000 nm band are cross-correlated. The SF wave will be mixed with the SC in the region of 400 nm ~ 444 nm, which will cause erroneous measurement results. A pre-filter for the blue part of the SC can avoid the mixing, but the SC pulses’ profile is broken. In contrast, the spatial separation between signal and inputs makes our method facile to fulfill a complete measurement of broadband pulses. (III) Due to the nanometer scale of the NW, phase matching constraints can be relaxed. Thus the measurable wavelength band can be largely extended since all the frequency components can be resolved within the NLO material. The complexity brought in by an angle-dithered crystal is avoided, and the optical alignment does not have to be very precise in a waveguide based measurement, both of which result in a simplified system. However, if this method is used for free-space pulse characterization, it needs a pre-survey on the dispersion and nonlinearity of delivering fiber’s, for instance, numerical simulations or a series of measurements with different fiber taper lengths have to be done. As a whole, this system is of great potential in integrating optics as an ultra-broadband optical device, and it also provides a promissing platform for intact observation of molecules dynamics and local field enhancement in future.

## Additional Information

**How to cite this article**: Yu, J. *et al.* Frequency-resolved optical gating measurement of ultrashort pulses by using single nanowire. *Sci. Rep.* **6**, 33181; doi: 10.1038/srep33181 (2016).
